# Decimative Spectral Estimation with Unconstrained Model Order

**DOI:** 10.1155/2012/917695

**Published:** 2012-02-22

**Authors:** Stavroula-Evita Fotinea, Ioannis Dologlou, Stylianos Bakamidis, Theologos Athanaselis

**Affiliations:** Department of Voice and Sound Technology, Institute for Language and Speech Processing / “Athena” R.C., Artemidos 6 and Epidavrou, 151 25 Maroussi, 6 Artemidos Street and Epidavrou, 15125 Paradissos Amaroussiou, Greece

## Abstract

This paper presents a new state-space method for spectral estimation that performs decimation by any factor, it makes use of the full set of data and brings further apart the poles under consideration, while imposing almost no constraints to the size of the Hankel matrix (model order), as decimation increases. It is compared against two previously proposed techniques for spectral estimation (along with derived decimative versions), that lie among the most promising methods in the field of spectroscopy, where accuracy of parameter estimation is of utmost importance. Moreover, it is compared against a state-of-the-art purely decimative method proposed in literature. Experiments performed on simulated NMR signals prove the new method to be more robust, especially for low signal-to-noise ratio.

## 1. Introduction

Various applications in the field of digital signal processing, including speech processing [1] as well as spectroscopy, that is, quantification of NMR signals, are employing complex damped sinusoidal models in order to represent a signal as a sum of exponentially damped complex-valued sinusoids [2–8]. The generalized model we use is given by

(1) $$\begin{array}{cc} s\left(n\right) & =\begin{array}{c} \overset{p}{\underset{i=1}{\sum }}\left(b_{i}e^{j(\varphi_{0}+\varphi_{i})}\right)e^{(-d_{i}+j2pf_{i})n} \end{array}\\ & =\overset{p}{\underset{i=1}{\sum }}g_{i}z_{i}^{n},\;n=0,\ldots ,N-1, \end{array}$$

where *p* is the number of complex damped sinusoids that comprise the measured signal. The objective is to estimate the frequencies *f*_*i*_, damping factors *d*_*i*_, amplitudes *b*_*i*_, and phases *φ*_0_ + *φ*_*i*_, *i* = 1,…, *p*. *φ*_0_ is the zero order phase, whereas *φ*_*i*_ represents extra degrees of freedom.

The new method proposed here is called DESE_D (DEcimative Spectral Estimation by factor *D*), which can perform decimation by any factor and exploits the full data set, whereas it is not obliged to reduce the size of the Hankel matrix as *D* increases, allowing the use of size *N*/2 approximately. The advantage of DESE_D relies on the fact that it can benefit from the higher pole resolution obtained by decimation [9], while at the same time is not bound to use smaller sizes of Hankel matrices, as other decimative approaches are. The new method makes use of Singular Value Decomposition. The DESE_D is a generalization of the DESE2 method proposed in [10], which performs decimation by factor 2.

The new method has been tested and compared to TLS-ESPRIT and LS-ESPRIT, that lie among the most promising methods for parameter estimation solving the same overdetermined system of equations in a total least squares and least squares sense, respectively. Moreover, their decimative versions are being presented and compared with DESE_D for the same decimation factors. In addition, the new method has been tested against a purely decimative method existing in the literature. In the sections that follow the proposed DESE_D method as well as the methods against which it is tested are presented and the superior performance of DESE_D is shown through Monte-Carlo-based experiments. This can be explained from signal processing theory where it is proved that decimation increases spectral resolution as it brings the frequencies of the sinusoids further apart. The MonteCarlo technique is used to alleviate the random effects of the noisy poles. It is also expected the improvement due to decimation to be more important at low signal-to-noise ratio SNR.

Note that the subspace estimation method TLS-ESPRIT [11] that we are using does not act on the covariance matrix but on the corresponding data matrix, the latter presenting some numerical advantages. Similarly, we are using in this paper the LS-ESPRIT method [12], which is also acting on the data matrix in a Least Squares sense (instead of Total Least Squares). The same method in NMR literature is known as HSVD (Hankel Singular Value Decomposition) [13]. We use the names TLS-ESPRIT and LS-ESPRIT as more suitable for the signal processing the literature. Also note that the conventional decimation method we are referring to as CONDE_D [9, 14, 15] can be also seen as a decimated version of the ESPRIT. However, the TLS-ESPRIT and LS-ESPRIT decimate in a different way than CONDE_D, according to the principles introduced for the DESE_D method.

Section 2.1 introduces DESE and presents a derivation for the decimation factor *D* case.  Section 2.2 contains the algorithmic presentation for DESE_D, while special cases are discussed in Section 2.3. The relation between DESE_D and a previously proposed conventional decimative spectral estimation method (CONDE_D) along with its algorithmic presentation is described in Section 2.4. In Section 3 aspects regarding the decimative versions of two existing spectral estimation methods the TLS-ESPRIT and LS-ESPRIT methods are presented. More specifically, the TLS-ESPRIT algorithm is briefly presented in Section 3.1, while its decimative version called TLS-ESPRIT_D is shown in Section 3.2. Similarly, Section 3.3 presents the LS-ESPRIT algorithm and Section 3.4 its decimative version, called LS-ESPRIT_D. In Section 3.5 computational considerations for DESE_D versus the other methods are discussed. Experimentation and results are found in Section 4, while concluding remarks follow in Section 5.

## 2. The DESE_D Method

### 2.1. Derivation

In [10] we have presented a derivation for the DESE_2 (decimation factor 2) method. A different derivation by employing the well-known Vandermonde decomposition as well as generalization to the decimation factor *D* case is presented in this paper.

Let *S*_*H*_ be the *L* × *M* Hankel signal observation matrix of our deterministic signal of *p* exponentials *s*(*n*), *n* = 0,…, *N* − 1

(2) $$S_{H}=\left[\begin{array}{cccc} {\tilde{s}}_{0} & {\tilde{s}}_{1} & \cdots & {\tilde{s}}_{M-1} \end{array}\right]$$

with *L* − *D* ≤ *M*,  *p* < *L* − *D* and *L* + *M* − 1 = *N*. Note that ${\tilde{s}}_{n}$ are the column vectors of *S*_*H*_, for *n* = 0, 1,…, *M* − 1. In particular ${\tilde{s}}_{n}^{T}=\left[s\left(n\right),\;s\left(n+1\right),\ldots ,s\left(n+L-1\right)\right]$, with *s*(*n*) being the *n*th sample of the signal *s*. Let the *L* − *D* × *M* matrices *S*↓_*D*_ and *S*↑_*D*_ be the *D* order lower shift (top *D* rows deleted) and the *D* order upper shift (bottom *D* rows deleted) equivalents of *S*_*H*_. The best choice for L and M is discussed in Section 2.2.

**Theorem 1**

Assuming that the signal s in (1) has unique poles (multiplicity one) and it is noise free, there is an (*L* − *D*) order matrix *X*, such that,

(3) $$XS{↑}_{D}\;=\;S{↓}_{D}$$

and all the signal's decimated poles (i.e., the poles of the signal multiplied by the factor *D*), are equal to the nonzero eigenvalues of *X*.

A solution of (3) is given by *X* = *S*↓_*D*_  pinv(*S*↑_*D*_) and contains the decimated poles of the signal where pinv denotes the Moore-Penrose pseudoinverse.

**Proof**

The first claim of the theorem is true because *S*_*H*_ is constructed of *p* complex damped sinusoids and therefore any row of *S*↓_*D*_ can be written as a linear combination of the rows of *S*↑_*D*_.

We want then to prove that *p* of the eigenvalues of the matrix

(4) $$X=S{↓}_{D}\;\text{pinv}\left(S{↓}_{D}\right)$$

are equal to the decimated signal pole estimates *z*_*i*_^*D*^, *i* = 1,…, *p*.

Let us consider the well-known Vandermonde decomposition of *S*_*H*_ *:*

(5) $$S_{H}=AGB^{T},$$

where superscript *T* denotes transpose and the *L* × *p* matrix *A*, *p* × *p* matrix *G* and *M* × *p* matrix *B* are defined as follows:

(6) $$\begin{array}{c} A=\left(\alpha_{H}\left(z_{1}\right)\cdots \alpha_{H}\left(z_{p}\right)\right),\;\;a_{H}\left(z\right)={\left(z^{0}z^{1}\cdots \;z^{L-1}\right)}^{T},\\ B=\left(b_{H}\left(z_{1}\right)\cdots b_{H}\left(z_{p}\right)\right),\;\;b_{H}\left(z\right)={\left(z^{0}z^{1}\cdots \;z^{M-1}\right)}^{T},\\ G=\mathrm{diag}\left(g_{1},\ldots ,g_{p}\right). \end{array}$$

We can then easily write *S*↓_*D*_ = *A*↓_*D*_ *GB* ^*T*^ and *S*↑_*D*_ = *A*↑_*D*_ *GB* ^*T*^ where *A*↓_*D*_ and *A*↑_*D*_ are defined from *A*, similarly to the way *S*↓_*D*_ and *S*↑_*D*_ are defined from *S*_*H*_.

Hence, (3) can be written as

(7) $$\left(XA{↑}_{D}-A{↓}_{D}\right)GB^{T}=0\Leftrightarrow XA{↑}_{D}=A{↓}_{D}.$$

The latter system of linear equations has an infinite number of solutions given by

(8) $$X=A{↓}_{D}{\left(A^{H}{↑}_{D}A{↑}_{D}\right)}^{-1}A^{H}{↑}_{D}+\Delta^{H},$$

where superscript *H* denotes Hermitian conjugate and Δ^*H*^ *A*↑_*D*_ = 0. Let us consider now the *p* × *p* diagonal matrix Φ_*D*_ containing the decimated signal poles *z*_*i*_^*D*^, *i* = 1,…, *p*. It is easy then to see that *A*↓_*D*_ = *A*↑_*D*_Φ_*D*_. Moreover, *A*↑_*D*_ = *A*↓_*D*_Φ_D_^−1^ resulting in Δ^*H*^ *A*↓_*D*_ = 0.

Hence, since ||*X*||_*F*_^2^ = ||*X*_0_||_*F*_^2^ + ||Δ^*H*^||_*F*_^2^ (||·||_*F*_ denotes the Frobenius norm), the minimum-norm solution to (7) which we compute is

(9) $$X_{0}=\;A{↓}_{D}\;{\left(A^{H}{↑}_{D}A{↑}_{D}\right)}^{-1}A^{H}{↑}_{D}.$$

Furthermore, knowing that *A*↓_*D*_ = *A*↑_*D*_  Φ_*D*_ it is also valid that *A* ^*H*^↑_*D*_ *A*↓_*D*_ = *A* ^*H*^↑_*D*_ *A*↑_*D*_Φ_*D*_ which leads to (*A* ^*H*^↑_*D*_ *A*↑_*D*_)^−1^ *A* ^*H*^↑_*D*_ *A*↓_*D*_ = Φ_*D*_.

By setting *W* = *A*↓_*D*_ and *Y* = (*A* ^*H*^↑_*D*_ *A*↑_*D*_)^−1^ *A* ^*H*^↑_*D*_ it is straightforward to see that *X*_0_ = *WY* and Φ_*D*_ = *YW*. According to the theorem in [16] the nonzero eigenvalues of *WY* are equal to the nonzero eigenvalues of YW. Consequently, the *p* nonzero eigenvalues of *X*_0_(rank⁡(*A*↑_*D*_) = *p*) are equal to the eigenvalues of Φ_*D*_, that is, the decimated poles.

The novelty of the above theorem relies on the generalization of the simpler problem for *D* = 1 as described in [17] to any factor *D*. In general, decimation may introduce aliasing effects that one should take into account in the algorithm. This is easy to do when prior knowledge (exact or approximate) for the frequencies of the complex damped sinusoids is available. In this case, one can undo the effects of aliasing that might occur when high decimation is used by employing filtering techniques (as described in [18]) to extract the useful sinusoids prior to estimating their parameters. Alternatively, when the frequencies are clustered together one faces the so-called “high resolution” scenario described in [9].

**The Proposed Method in the Presence of Noise**

In case of real life signals (i.e., signals impaired with additive noise) the peaks are embedded in noise and the rank of matrix *S*_*H*_ is full. The equality (3) does not hold any longer because the signal does not obey linear models. If the number of complex peaks to estimate is *p*, the matrix *S*↑_*D*_ can be enhanced by reducing its rank to *p*.

To do so we employ the SVD of *S*↑_*D*_ and we retain the *p* largest singular values based on the assumption that the noise energy is lower than the energy of the *p* sinusoids of the signal *s*. The resulting matrix *S*↑_*D*_ *e* has rank *p*. Then *X* is computed from *XS*↑_*D*_ *e* ≈ *S*↓_*D*_ which gives rise to an overdetermined system of equations with the following solution *X* = *S*↓_*D*_ pinv(*S*↑_*D*_ *e*).

Note that since matrix *S*↑_*D*_ *e* has rank *p*, *X* is also of rank *p* (minimum of ranks of *S*↑_*D*_ *e* and *S*↓_*D*_) and this guarantees that only *p* of the eigenvalues of *X* is nonzero and corresponds to the decimated signal pole estimates. This yields the desired decimated estimates of frequencies and damping factors from the angles and magnitudes, respectively, of the eigenvalues of *X*. These decimated estimates are converted to their nondecimated equivalents *f*_*i*_ (frequency estimates) and *d*_*i*_ (damping factor estimates) and a computation in a total least squares sense of estimates *g*_*i*_ then takes place. In this way, complex-valued linear parameter estimates of *g*_*i*_ are calculated, from which amplitude *b*_*i*_ and phases *φ*_0_ + *φ*_*i*_ estimates are determined as the magnitudes and angles of *g*_*i*_, respectively.

### 2.2. DESE_D Algorithmic Presentation

Let *S*_*H*_ be the *L* × *M* Hankel signal observation matrix of our deterministic signal of *p* exponentials *s*(*n*), *n* = 0,…, *N* − 1, with *L* − *D* ≤ *M*, *p* < *L* − *D* and *L* + *M* − 1 = *N*, where *D* denotes the decimation factor.

The proposed algorithm, involves the following five steps.

**Step 1 (DESE_D)**

We compute the *L* × *M* matrix *S*_*H*_ of (2) from the *N* data points *s*(*n*) of (1).

**Step 2 (DESE_D)**

We compute the *S*↓_*D*_ and *S*↑_*D*_ as the *D* order lower shift (top *D* rows deleted) and the *D* order upper shift (bottom *D* rows deleted) equivalents of *S*_*H*_.

The best results are obtained when we use the (*L* − *D*) × *M* matrices *S*↓_*D*_ and *S*↑_*D*_ as square as possible [11, 19–21].

**Step 3 (DESE_D)**

We compute the enhanced version *S*↑_*D*_ *e* of *S*↑_*D*_ in the following way. We employ the SVD of *S*↑_*D*_, *S*↑_*D*_ = *U*↑_*D*_Σ↑_*D*_ *V*↑_*D*_^*H*^ and we truncate to order *p* by retaining only the largest *p* singular values.

**Step 4 (DESE_D)**

We compute matrix *X* = *S*↓_*D*_ pinv(*S*↑_*D*_ *e*).

The eigenvalues ${\hat{\lambda }}_{i}$ of *X* give the decimated signal pole estimates, which in turn give the estimates for the damping factors and frequencies of (1).

**Step 5 (DESE_D)**

The last step is to compute the phases and the amplitudes. This is done by finding a least squares solution to (1), with *z*_*i*_ replaced by the estimates and *s*(*n*) given by the signal data points.

Matrix *X* of Step 4 in the above described version of DESE_D, is computed in a least squares sense. We could however, compute matrix *X* in a total least squares sense using the Theorem 3.10 presented in [17]. We can, hence, obtain the DESE_D_TLS method, presented in [22], where the obtained results suggest that DESE_D and DESE_D_TLS perform rather similarly for small noise standard deviations, whereas DESE_D seems slightly more robust for large noise standard deviations than its total squares counterpart. This is the reason why only DESE_D was included in the experimentation reported here.

### 2.3. DESE_D Special Cases

The above presented method can also serve as a state-space method for spectral estimation, if seen and implemented with no decimation whatsoever (*D* = 1). In this case, matrices *S*↓_1_ and *S*↑_1_ are, respectively, the first-order lower shift (top row deleted) and first-order upper shift (bottom row deleted) of the original Hankel *S*_*H*_ of (2) with *L* − 1 ≤ *M*, *p* < *L* − 1 and *L* + *M* − 1 = *N*.

A variation of such a nondecimative method, called CSE, was proposed in [23]. In this case both matrices *S*↓_1_ and *S*↑_1_ (of Step 2) were enhanced (truncated to order *p*) with the use of SVD. Thus, Steps 3 and 4 presented above are replaced by the following step.

**Step 3 (a)**

We compute the enhanced version *S*↓_1_ *e* of *S*↓_1_ in the following way. We employ the SVD of *S*↓_1_, *S*↓_1_ = *U*↓_1_Σ↓_1_ *V*↓_1_^*H*^  and we truncate to order *p* by retaining only the largest *p* singular values.

In the same way, we compute the enhanced version *S*↑_1_ *e* of *S*↑_1_.

**Step 4 (a)**

We compute matrix *X* = (*S*↓_1_ *e*)pinv(*S*↑_1_ *e*).

The eigenvalues ${\hat{\lambda }}_{i}$ of *X* give the signal pole estimates, which in turn give the estimates for the damping factors and frequencies of (1). Note that the CSE method was also proven to be more robust in terms of bad runs when compared to TLS-ESPRIT; however, compared to DESE_1, similar results were obtained while the complexity was increased due to the second enhancement.

When only one enhancement is performed (to matrix *S*↑_1_), the nondecimative method (*D* = 1, for DESE_D) is identical to a method proposed in [24], the MATPEN method.

For MATPEN(=DESE_1), Step 4(a) is replaced by *X* = *S*↓_1_pinv(*S*↑_1_ *e*).

The eigenvalues ${\hat{\lambda }}_{i}$ of *X* give the signal pole estimates, which in turn give the estimates for the damping factors and frequencies of (1).

### 2.4. DESE_D versus Other Decimative Methods

The drawbacks of conventional decimative methods are related to the size of the data set and to the overdetermined model order that can be used. Already proposed decimative methods (e.g., [9]), even though they make use of the full data set available, are obliged to reduce the maximum possible matrix size as *D* increases. Hence, they relate the size of the Hankel matrix *n* with *D*, according to *n* = *N*/(2*D*).

This implies that the efficiency of the overdetermined model is reduced. On the contrary, DESE_D does not present this drawback and allows the use of matrix size *n* = (*N* + 1)/2 − *D*/2, that change very slowly with respect to decimation factor *D*.

The DESE_D has been tested against a decimation method proposed in [9, 14, 25], which we call below CONDE_D (CONventional DEcimative method for decimation factor *D*).

The method makes use of the auto- and cross-covariance matrices of the input signal, and decimated sequences of the input signal. Then, averaged covariance matrices are used for parameter estimation of the complex damped sinusoids. Next, it employs Singular Value Decomposition of the resulting matrix to truncate to order *p* and proceeds with estimation of the frequency and damping factor in a total least squares sense.

The method's algorithmic presentation for decimation factor *D* involves the following five steps.

**Step 1 (CONDE_D)**

We compute the *L* × *M*  (*L* = *M* = *N*/(2*D*)) Hankel matrix *C*_*k*_ that corresponds to the *k*th decimated signal, *c*_*k*_(*n*) = *s*(*k* : *D* : *N*), where *D* is the decimation factor, from the *N* data points *s*(*n*) of (1).

**Step 2 (CONDE_D)**

We compute a global matrix *C* by concatenating *C*_*k*_, *k* = 1,…, *D* as shown below:

(10) $$C\;=\left[C_{1}\;:\;C_{2}\;:\;\cdots \;:\;C_{D}\right].$$

We then compute a global covariance matrix *R* = *CC* ^*H*^.

**Step 3 (CONDE_D)**

We compute the eigen analysis of *R* = *U*Λ*U* ^*H*^ to deduce *U*, which in turn is truncated to order *p*, thus, yielding *U*_*p*_.

**Step 4 (CONDE_D)**

We compute the solution *Q* of *U*↑_*p*_ *Q* = *U*↓_*p*_, in a total least squares sense, where *U*↓_*p*_(*U*↑_*p*_) are derived from *U*_*p*_ by deleting its top (bottom) row. The eigenvalues ${\hat{\lambda }}_{i}$ of *Q* give the decimated signal pole estimates, which in turn give the estimates for the damping factors and frequencies of (1).

**Step 5 (CONDE_D)**

The last step is to compute the phases and the amplitudes. This is done by finding a least squares solution to (1), with *z*_*i*_ replaced by the estimates and *s*(*n*) given by the signal data points.

## 3. Decimative Versions of TLS-ESPRIT and LS-ESPRIT

The new concept of using all data samples available while practically imposing no constraints between the size of HANKEL matrix and decimation factor *D*, included in the DESE_D method, can be implemented in other state-space spectral estimation methods, thus, deriving a new family of methodologies.

The subsections that follow present the TLS-ESPRIT and LS-ESPRIT methods along with their decimative versions TLS-ESPRIT_D and LS-ESPRIT_D, respectively.

### 3.1. The TLS-ESPRIT Algorithm

The TLS-ESPRIT method, reported in [11], consists of using the Hankel matrix, performing an SVD decomposition and reducing the size of matrices to order *p*. Damping factors *d*_*i*_ and the frequencies *f*_*i*_ are estimated in a total least squares sense. Phases and amplitudes are estimated using the least squares method.

**Step 1 (TLS-ESPRIT)**

We compute the SVD of the *L* × *M* Hankel matrix *S*_*H*_ of (2) from the *N* data points *s*(*n*) of (1):

(11) $$S_{H}=U_{L\times L}\Sigma_{L\times M}V_{M\times M}^{H},$$

where *L* ≤ *M*. The best results are obtained when we use *L* = *M*(+1) = *N*/2.

**Step 2 (TLS-ESPRIT)**

We truncate *U*, Σ, *V* to order *p* and compute *S*_*p*_ = *U*_*p*_Σ_*p*_ *V*_*p*_^*H*^ where *U*_*p*_,  Σ_*p*_, *V*_*P*_  are the first *p* columns of *U*_*L*×*L*_, Σ_*L*×*M*_, *V*_*M*×*M*_^*H*^.

**Step 3 (TLS-ESPRIT)**

We compute the solution *Q* of *U*↑_*p*_ *Q* = *U*↓_*p*_, in a total least squares sense, where *U*↓_*p*_(*U*↑_*p*_) are derived from *U*_*p*_ by deleting its top (bottom) row. The eigenvalues ${\hat{\lambda }}_{i}$ of *Q* give the signal pole estimates, which in turn give the estimates for the damping factors and frequencies of (1).

**Step 4 (TLS-ESPRIT)**

The last step is to compute the phases and the amplitudes. This is done by finding a least squares solution to (1), with *z*_*i*_ replaced by the estimates and *s*(*n*) given by the signal data points. It is worth noting that TLS-ESPRIT and CONDE_1 (no decimation whatsoever) are identical.

### 3.2. The TLS-ESPRIT_D Algorithm

By using the notion introduced by DESE_D that implies minor reduction of the Hankel matrix size with respect to decimation factor *D*, the decimative version of TLS-ESPRIT can be easily derived. More precisely, appropriate formation of matrix *U*_*p*_—similar to that of matrix *S*_*H*_ in the proposed DESE_D-, creates the TLS-ESPRIT_D decimative version for decimation factor *D*. Its algorithmic presentation is shown below. Please note that Steps 1and 2 are identical in the two approaches.

**Step 1 (TLS-ESPRIT)**

We compute the SVD of the *L* × *M* Hankel matrix *S*_*H*_ of (2) from the *N* data points *s*(*n*) of (1):

(12) $$S_{H}=U_{L\times L}\Sigma_{L\times M}V_{M\times M}^{H},$$

where *L* ≤ *M*. The best results are obtained when we use *L* = *M*(+1) = *N*/2.

**Step 2 (TLS-ESPRIT)**

We truncate *U*, Σ, *V* to order *p* and compute: *S*_*p*_ = *U*_*p*_Σ_*p*_ *V*_*p*_^*H*^ where *U*_*p*_,  Σ_*p*_,  *V*_*p*_ are the first *p* columns of *U*_*L*×*L*_, Σ_*L*×*M*_, *V*_*M*×*M*_^*H*^.

**Step 3 (TLS-ESPRIT_D)**

We compute the solution *Q* of *U*↑_*D*_ *Q* = *U*↓_*D*_, in a total least squares sense, where *U*↓_*D*_(*U*↑_*D*_) are derived from *U*_*P*_ by deleting its top *D* (bottom *D*) rows, respectively. The eigenvalues ${\hat{\lambda }}_{i}$ of *Q* give the decimated signal pole estimates, which in turn give the estimates for the damping factors and frequencies of (1).

**Step 4 (TLS-ESPRIT_D)**

The last step is to compute the phases and the amplitudes. This is done by finding a least square solution to (1), with *z*_*i*_ replaced by the estimates and *s*(*n*) given by the signal data points. Note that in [26], a different decimated version of TLS-ESPRIT (=HTLS in NMR literature) is presented, which is not treated in this paper.

### 3.3. The LS-ESPRIT Algorithm

If instead of computing in a total least squares sense the solution *Q* of *U*↑_*p*_ *Q* = *U*↓_*p*_, one employs the least squares solution, one uses LS-ESPRIT [12].

In this case, Step 3_TLS-ESPRIT of the Section 3.1 above is replaced by the following.

**Step 3 (LS-ESPRIT)**

We compute the solution *Q* of *U*↑_*p*_ *Q* = *U*↓_*p*_, in a least squares sense, where *U*↓_*p*_(*U*↑_*p*_) are derived from *U*_*p*_ by deleting its top (bottom) row. Hence, *Q* = pinv(*U*↑_*P*_)*U*↓_*P*_.

The eigenvalues ${\hat{\lambda }}_{i}$ of *Q* give the signal pole estimates, which in turn give the estimates for the damping factors and frequencies of (1).

### 3.4. The LS-ESPRIT_D Algorithm

One can easily derive LS-ESPRIT_D, as it was done for TLS-ESPRIT_D, by appropriate formation of matrix *U*_*p*_ for decimation factor *D* and by solving in a least squares sense for matrix *Q*.

In this case, Step 3_TLS-ESPRIT_D of the Section 3.2 above is replaced by the following.

**Step 3 (LS-ESPRIT_D)**

We compute the solution *Q* of *U*↑_*D*_ *Q* = *U*↓_*D*_, in a least squares sense, where *U*↓_*D*_  (*U*↑_*D*_) are derived from *U*_*p*_ by deleting its top *D* (bottom *D*) rows, respectively. Hence, *Q* = pinv(*U*↑_*D*_)*U*↓_*D*_. The eigenvalues ${\hat{\lambda }}_{i}$ of *Q* give the decimated signal pole estimates, which in turn give the estimates for the damping factors and frequencies of (1).

### 3.5. Computational Considerations for DESE_D

Regarding the computational complexity, DESE_D involves one large SVD (singular value decomposition) and one large EVD (eigenvalue decomposition). Note that the pinv operation in Step 4 of the DESE_D algorithm is of no computational load, since the SVD of the matrix involved in the pseudoinverse operation is already available from the previous step (Step 3_DESE_D). Moreover, only *p* of the eigenvalues of matrix *X* involved in the EVD, are nonzero, which can considerably reduce the complexity of the large EVD in DESE_D if a fast algorithm is used. On the other hand, the TLS-ESPRIT_D/LS-ESPRIT_D decimative versions require one large SVD and one small EVD. Consequently the difference of the complexity of DESE_D versus TLS-ESPRIT_D/LS-ESPRIT_D cannot be considered as a drawback for DESE_D. Regarding the complexity of CONDE_D, the fact that it uses lower dimension Hankel matrices (as decimation increases) improves its computational characteristics with respect to the other methods.

## 4. Experimental Results

All methods, namely, DESE_D, CONDE_D, LS-ESPRIT_D, and TLS-ESPRIT_D have been tested via simulations on a typical two peak reference signal, and two typical ^31^P NMR signals, in order to evaluate both robustness and accuracy of parameter estimation in the problem defined by (1). All the experiments have been conducted using the Matlab software.

The first signal is a two-peak signal often used in the literature (reference signal), the exact parameter values of which are presented in Table 1, while the sampling frequency is considered 1.

The second signal is a representative example simulating a typical ^31^P NMR signal of perfused rat liver. This ^31^P NMR signal comprises a fifth-order model function given in Table 2 by which *N* data points uniformly sampled at 10 KHz are exactly modeled.

Moreover, the third signal is also a representative example simulating a typical ^31^P NMR signal which comprises an eleventh-order model function given in Table 3 by which *N* data points uniformly sampled at 3 KHz are exactly modeled.

The data points of all signals are perturbed by white Gaussian noise whose real and imaginary components have standard deviation *σ*_*v*_.

Root mean-squared errors of the estimates of all signal parameters are computed using either 500 noise realizations or 3000 noise realizations (excluding *failures*) for different noise levels. A failure occurs when *not all peaks *are resolved within specified intervals lying symmetrically around the exact frequencies and when the estimated damping factors are nonpositive.

**Figure 1:**
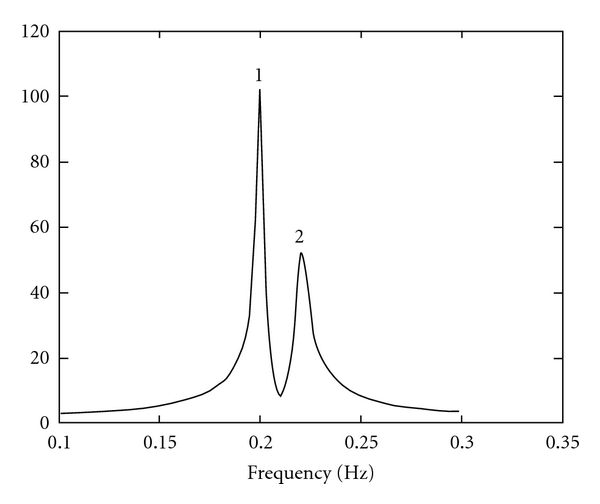
Fast Fourier transform (magnitude) of the two peak signal.

For the two peak reference signal (Figure 1), the half-widths of the intervals are, respectively, 0.0094 Hz and 0.0106 Hz and were deduced from the Cramer-Rao lower bounds of the two peaks at the noise standard deviation where these intervals are touching each other. These values are only used to determine when a failure (bad run) occurs. They depend on the signal parameters and the noise energy and show how far one can go before the two peaks cannot be resolved.

**Figure 2:**
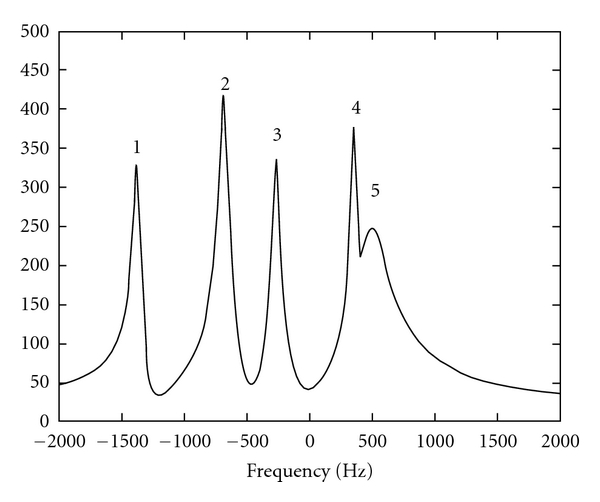
Fast Fourier transform (magnitude) of the five peak simulated ^31^P NMR signal.

For the five-peak ^31^P NMR signal (Figure 2), the half-widths of the intervals are, respectively, 82, 82, 82, 43, and 82 Hz, the values being derived from the Cramer-Rao lower bounds of the closest peaks 4 and 5 at the noise standard deviation where these intervals are touching each other. The number of complex damped sinusoids to be estimated is set to 5. The Cramer-Rao lower bounds are derived from the exact parameter values and *σ*_*v*_.

**Figure 3:**
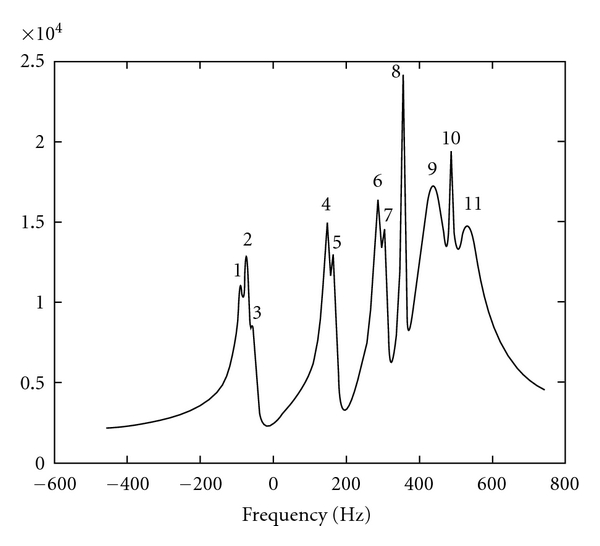
Fast Fourier transform (magnitude) of the eleven peak simulated ^31^P NMR signal.

For the eleven peak ^31^P NMR signal (Figure 3), the half-widths of the intervals are 8.6, 7.3, 8.6, 3.2, 3.2, 3.4, 3.6, 7.4, 5.5, 2.3, and, 7.7 Hz, for peak 1 to 11, respectively. These values are derived from the Cramer-Rao lower bounds of the closest peaks 4 and 5 at the noise standard deviation where these intervals are touching each other.

Comparative results between all methods are presented below for different noise standard deviations.

For all methods, we have used *N* = 128 and *M* = *N*/2 = 64 except for CONDE_D, for which *M* = 32(21) for *D* = 2(3), respectively.

**Figure 4:**
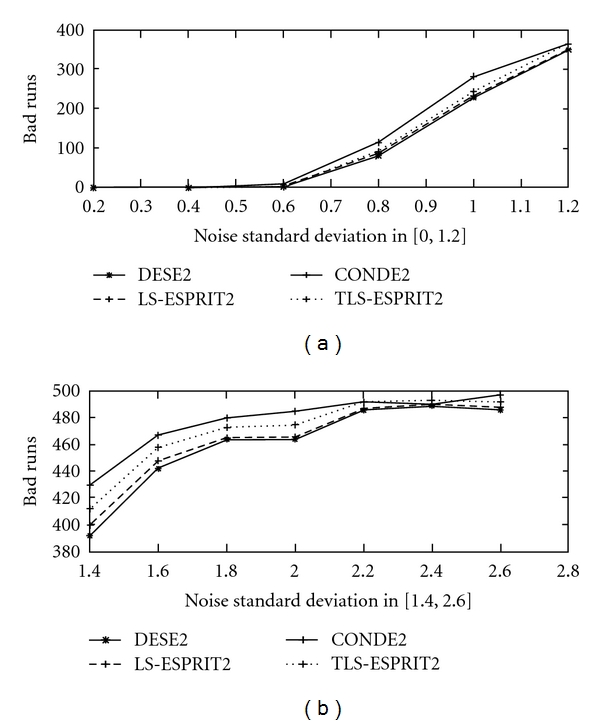
Number of Bad runs in 500 realisations for *σ*_*v*_ ∈ (0, 2.6] for the two-peak reference signal for methods DESE2, LS-ESPRIT2, TLS-ESPRIT2 and CONDE2.

In Figure 4 failure rates (bad runs) in 500 realizations are depicted as a function of noise standard deviation for the two peak reference signal. In this graphical representation, results are presented for methods DESE2, LS-ESPRIT2, TLS-ESPRIT2, and CONDE2.

**Figure 5:**
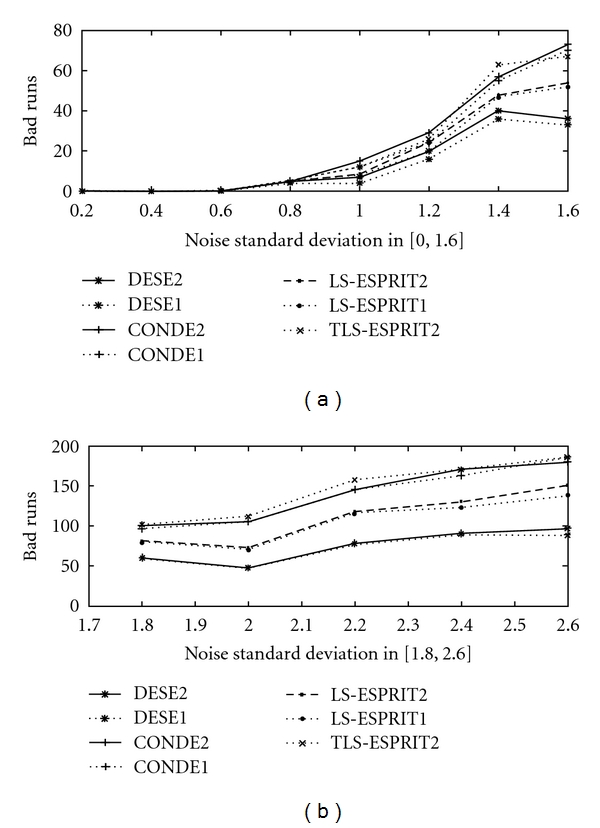
Number of Bad runs in 500 realisations for *σ*_*v*_ ∈ (0, 2.6] for the eleven peak simulated ^31^P NMR signal for methods DESE2, DESE1, CONDE2, CONDE1, LS-ESPRIT2, LS-ESPRIT1, and TLS-ESPRIT2.

The same quantity, namely, number of bad runs in 500 realizations, is depicted in Figure 5 for the eleven peak simulated ^31^P NMR signal, for methods DESE2 & 1, CONDE2 & 1, LS-ESPRIT2 & 1, and TLS-ESPRIT2.

Note that DESE2 and DESE1 have fewer bad runs than the other methods under consideration. This is even more evident as the noise increases.

Moreover, we have conducted further experiments to improve the statistical behavior of RMSE figures when the number of bad runs tends to be very large. We decided to increase considerably the number of Monte Carlo trials to insure that the number of good runs that are taken into account to deduce the RMSE is big enough.

**Figure 6:**
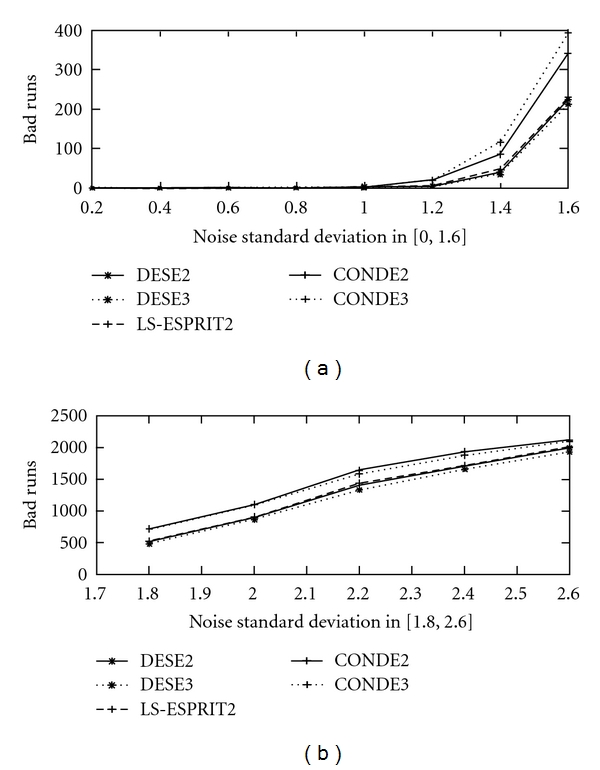
Number of bad runs in 3000 realisations for *σ*_*v*_ ∈ (0, 2.6] for the five peak simulated ^31^P NMR signal for methods DESE2, DESE3, LS-ESPRIT2, CONDE2 and CONDE3.

**Figure 7:**
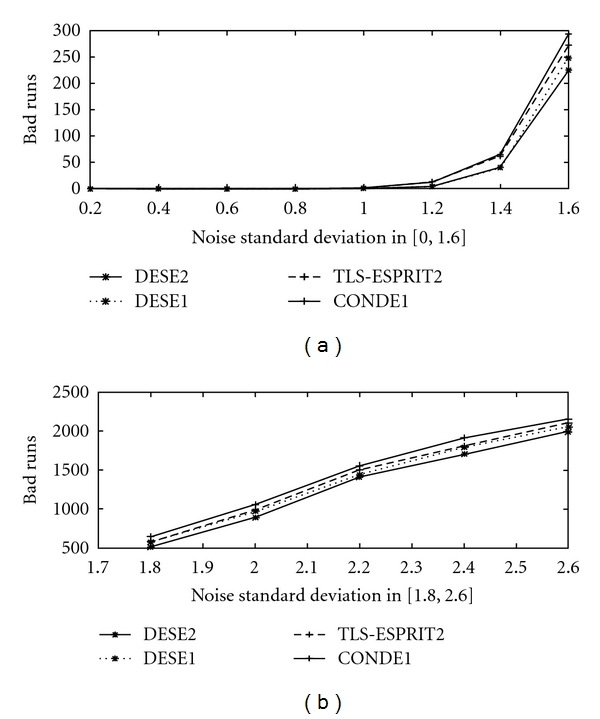
Number of bad runs in 3000 realisations for *σ*_*v*_ ∈ (0, 2.6] for the five peak simulated ^31^P NMR signal for methods DESE2, DESE1, TLS-ESPRIT2 and CONDE1 (=TLS-ESPRIT).

In Figure 6 the number of bad runs for 3000 trials are presented for the five peak simulated ^31^P NMR signal, for *σ*_*v*_∈(0, 2.6], for methods DESE2, DESE3, LS-ESPRIT2, CONDE2, and CONDE3. The same quantity is depicted in Figure 7 for methods DESE2, DESE1, TLS-ESPRIT2, and CONDE1 (=TLS-ESPRIT).

Note that here again DESE2 and DESE3 present fewer bad runs than the other methods, which becomes more evident as the noise increases. In general, DESE3 performs better in terms of robustness than DESE2 as expected, because it brings the peaks even more further apart due to the decimation factor 3 instead of 2, while the matrix size remains practically unchanged.

**Figure 8:**
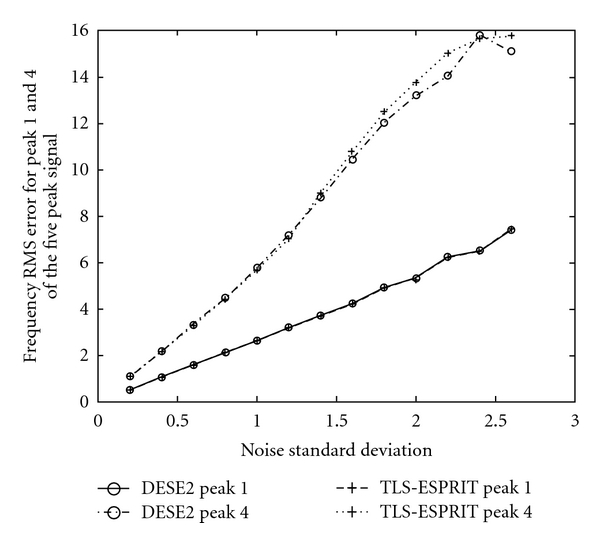
Frequency root mean square errors in 3000 realisations for Peaks 1 and 4 of the five peak simulated ^31^P NMR signal, for methods DESE2 & TLS-ESPRIT and for *σ*_*v*_ ∈ (0,2.6].

In Figure 8 root mean square errors of the frequency estimates are presented for DESE2 and TLS-ESPRIT and for *σ*_*v*_∈(0, 2.6], for peaks 1 and 4 of the five peak simulated ^31^P NMR signal, for 3000 trials. Peak 4 of this signal is considered the most difficult to estimate since it is relatively close to peak 5.

**Figure 9:**
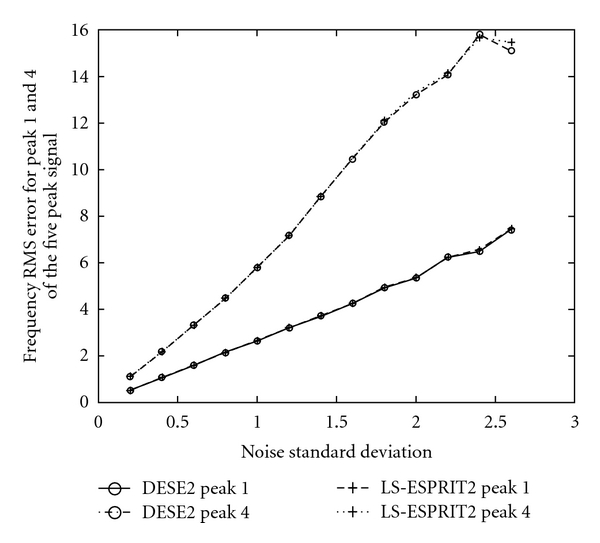
Frequency root mean square errors in 3000 realisations for Peaks 1 and 4 of the five peak simulated ^31^P NMR signal, for methods DESE2 & LS-ESPRIT2 and for *σ*_*v*_ ∈ (0,2.6].

**Figure 10:**
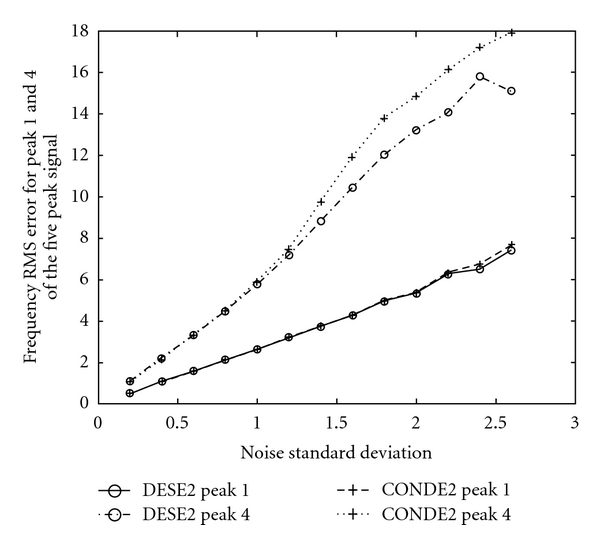
Frequency root mean square errors in 3000 realisations for Peaks 1 and 4 of the five peak simulated ^31^P NMR signal, for methods DESE2 & CONDE2 and for *σ*_*v*_ ∈ (0,2.6].

**Figure 11:**
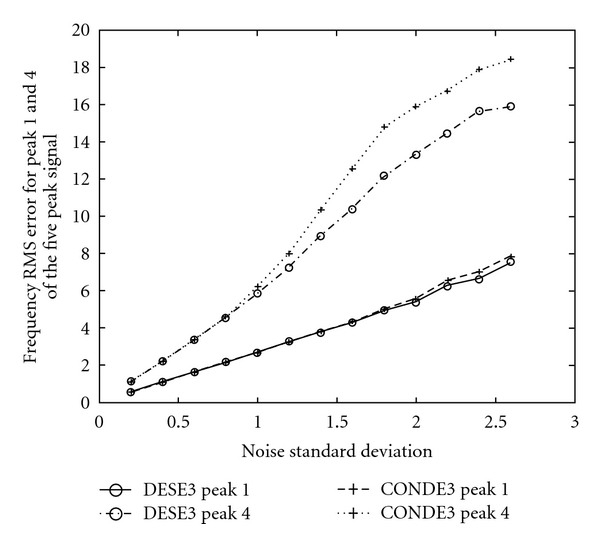
Frequency root mean square errors in 3000 realisations for Peaks 1 and 4 of the five peak simulated ^31^P NMR signal, for methods DESE3 & CONDE3 and for *σ*_*v*_ ∈ (0,2.6].

The same quantities (also for 3000 trials) for methods DESE2 versus LS-ESPRIT2 are presented in Figure 9, for DESE2 versus CONDE2 in Figure 10, and for methods DESE3 versus CONDE3 in Figure 11.

These graphs show the same trend, according to which, the DESE_D technique outperforms the other methods for both peaks 1 and 4, especially for low SNR. In particular, the difference in performance is more evident in the case of peak 4, which is more difficult to estimate due to its short distance from peak 5. Figures 8 to 11 show that DESE_D has always better or at least similar performance compared to the other techniques. More detailed results involving noise standard deviation, number of bad runs, and root mean-squared errors of frequency, damping factor, amplitude, and phase estimates for all signals are presented in tabular forms in [27].

It is worth noting that the increased number of trials improves the statistical behavior of the RMSE variance.

The above results suggest in all cases that the DESE_D approach performs similarly to the other methods for high *S*/*N* ratio. However, for low *S*/*N* ratio, despite the similarity of the root mean-squared errors of the estimated parameters, the DESE_D technique performs better due to its lower failure rate.

Note that the calculation of the root mean-squared error does not take into account failures and it is normal that methods with small number of bad runs will present larger error than those with big number of bad runs.

## 5. Conclusion

In this paper DESE_D, a new state-space decimative method, for spectral estimation was presented. It makes use of decimation by any factor *D* and SVD, to estimate frequencies, damping factors, amplitudes, and phases of complex damped sinusoids. DESE_D makes use of the full data set available and, unlike conventional decimation methods, it imposes no constraints to the size of the Hankel matrix, as decimation increases. It was tested in spectroscopy, one of the most demanding applications of digital signal processing in terms of accuracy. DESE_D was compared to a state-of-the-art decimative method, along with other state-of-the-art nondecimative ones together with their derived decimative counterparts. Examples on a two-peak reference signal as well as on two typical ^31^P NMR signals were presented and it was shown that DESE_D performs better than the other methods, especially for low signal-to-noise ratio.

## Figures and Tables

**Table 1:** Exact parameter values of the standard two peak reference signal with *φ*_*i*_ = 0.

Peak *i*	*f*_*i*_ (Hz)	*d*_*i*_ (rad/s)	*b*_*i*_	*ψ*_*i*_^(a)^
1	0.2	0.01	1	0
2	0.22	0.02	1	0

^(a)^ *ψ*_*i*_ = *φ*_0_180*π* expresses the phase in degrees.

**Table 2:** Exact parameter values of the five peak simulated ^31^P NMR signal, modelled by (1) with *φ*_*i*_ = 0.

Peak *i *	*f*_*i*_ (Hz)	*d*_*i*_ (rad/s)	*b*_*i*_	*ψ*_*i*_^(a)^
1	−1379	208	6.1	15
2	−685	256	9.9	15
3	−271	197	6.0	15
4	353	117	2.8	15
5	478	808	17.0	15

^(a)^ *ψ*_*i*_ = *φ*_0_180*π* expresses the phase in degrees.

**Table 3:** Exact parameter values of the eleven peak simulated ^31^P NMR signal, modelled by (1) with *φ*_*i*_ = 0.

Peak *i*	*f*_*i*_ (Hz)	*d*_*i*_ (rad/s)	*b*_*i*_	*ψ*_*i*_^(a)^
1	−86	50	75	135
2	−70	50	150	135
3	−54	50	75	135
4	152	50	150	135
5	168	50	150	135
6	292	50	150	135
7	308	50	150	135
8	360	25	150	135
9	440	285.7	1400	135
10	490	25	60	135
11	530	200	500	135

^(a)^ *ψ*_*i*_ = *φ*_0_180*π* expresses the phase in degrees.

## References

[1] Kumaresan R, Tufts DW (1982). Estimating the parameters of exponentially damped sinusoids and pole-zero modeling in noise. *IEEE Transactions on Acoustics, Speech, and Signal Processing*.

[2] Kung SY, S.Arun K, V. Bhaskar Rao D (1983). Statespace and singular-value decomposition-based approximation methods for the harmonic retrieval problem. *Journal of the Optical Society of America A*.

[3] Stoica P, Moses R (1997 ). *Introduction to Spectral Analysis*.

[4] Stoica P, Moses RL, Friedlander B, Soderstrom T (1989). Maximum likelihood estimation of the parameters of multiple sinusoids from noisy measurements. *IEEE Transactions on Acoustics, Speech, and Signal Processing*.

[5] Papy JM, de Lathauwer L, van Huffel S (2009). Exponential data fitting using multilinear algebra: the decimative case. *Journal of Chemometrics*.

[6] Poullet JB, Sima DM, Van Huffel S (2008). MRS signal quantitation: a review of time- and frequency-domain methods. *Journal of Magnetic Resonance*.

[7] Markovsky I, Van Huffel S (2007). Overview of total least-squares methods. *Signal Processing*.

[8] Sandgren N, Selén Y, Stoica P, Li J (2004). Parametric methods for frequency-selective MR spectroscopy—a review. *Journal of Magnetic Resonance*.

[9] Stoica P, Nordsjö AE (1997). Subspace-based frequency estimation in the presence of moving-average noise using decimation. *Signal Processing*.

[10] Fotinea SE, Dologlou I, Carayannis G Decimation and SVD to estimate exponentially damped sinusoids in the presence of noise.

[11] Vanhuffel S, Chen H, Decanniere C, Van Hecke P (1994). Algorithm for time-domain NMR data fitting based on total least squares. *Journal of Magnetic Resonance A*.

[12] Roy R, Paulraj A, Kailath T (1986). ESPRIT—a subspace rotation approach to estimation of parameters of cisoids in noise. *IEEE Transactions on Acoustics, Speech, and Signal Processing*.

[13] Pijnappel WWF, van den Boogaart A, de Beer R, van Ormondt D (1992). SVD-based quantification of magnetic resonance signals. *Journal of Magnetic Resonance*.

[14] Halder B, Kailath T (1997). Efficient estimation of closely spaced sinusoidal frequencies using subspace-based methods. *IEEE Signal Processing Letters*.

[15] Stoica P, Li J, Tan X (2008). On spatial power spectrum and signal estimation using the Pisarenko framework. *IEEE Transactions on Signal Processing*.

[16] A. Horn Roger, R. Johnson Charles (1985). *Matrix Analysis*.

[17] Van Huffel S, Vandewalle J (1991). *The Total Least Squares Problem. Computational Aspects and Analysis*.

[18] Sundin T, Vanhamme L, Van Hecke P, Dologlou I, Van Huffel S (1999). Accurate quantification of ^1^H spectra: from FIR filter design for solvent suppression to parameter estimation. *Journal of Magnetic Resonance*.

[19] Dologlou I, Carayannis G (1991). LPC/SVD analysis of signals with zero modeling error. *Signal Processing*.

[20] Stoica P, Selén Y (2004). A review of information criterion rules. *IEEE Signal Processing Magazine*.

[21] Fotinea S-E, Dologlou I, Carayannis G (2006). On the use of decimation for efficient spectral estimation. *International Journal of Computer Mathematics and Its Applications*.

[22] Fotinea S-E, Dologlou I, Carayannis G, Van Huffel S, Lemmerling P (2002). A new decimative spectral estimation method with unconstrained model order and decimation factor. *Total Least Squares and Errors-in-Variables Modeling: Analysis, Algorithms and Applications*.

[23] Fotinea SE, Dologlou I, Hatzigeorgiu N, Carayannis G Spectral estimation based on the eigenanalysis of companion-like matrices.

[24] Hua Y, Sarkar TK (1990). Matrix pencil method for estimating parameters of exponentially damped/undamped sinusoids in noise. *IEEE Transactions on Acoustics, Speech, and Signal Processing*.

[25] Morren G, Lemmerling P, Van Huffel S (2003). Decimative subspace-based parameter estimation techniques. *Signal Processing*.

[26] Morren G, Lemmerling P, Van Huffel S Decimative subspace-based parameter estimation techniques applied to magnetic resonance spectroscopy signals.

[27] Fotinea S-E, Dologlou I, Carayannis G (2001). Detailed results for the work presented in “Decimative Spectral Estimation based on the eigenanalysis of Hankel matrices”. *ILSP Internal Report*.

